# Mitochondria in cutaneous health, disease, ageing and rejuvenation—the 3PM-guided mitochondria-centric dermatology

**DOI:** 10.1007/s13167-025-00400-z

**Published:** 2025-02-14

**Authors:** Olga Golubnitschaja, Nafiseh Sargheini, Janine Bastert

**Affiliations:** 1 Predictive, Preventive and Personalised (3P) Medicine, Department of Radiation Oncology, University Hospital Bonn, Rheinische Friedrich-Wilhelms-Universität Bonn, 53127 Bonn, Germany; 2 Max Planck Institute for Plant Breeding Research, Carl-Von-Linne-Weg 10, 50829 Cologne, Germany; 3 Private Dermatological Clinic, Kirchheimer Str. 71, 70619 Stuttgart, Germany

**Keywords:** Predictive Preventive Personalised Medicine (PPPM / 3PM), Dermatology, Onco-dermatology, Mito-dermatology, Aesthetic medicine, Mitochondria, Homoeostasis, Mitophagy, Skin, Health, Disease, Ageing, Rejuvenation, Vitiligo, Wound healing, Inflammation, Scar quality, Targeted prevention, CoQ10, Resveratrol, Quercetin, Individualised patient profile, Tailored treatments, Paradigm change, Cost-efficacy, Health policy

## Abstract

Association of both intrinsic and extrinsic risk factors leading to accelerated skin ageing is reflected in excessive ROS production and ir/reversible mitochondrial injury and burnout, as abundantly demonstrated by accumulating research data. Due to the critical role of mitochondrial stress in the pathophysiology of skin ageing and disorders, maintained (primary care) and restored (secondary care) mitochondrial health, rejuvenation and homoeostasis are considered the most effective holistic approach to advance dermatological treatments based on systemic health–supportive and stimulating measures. Per evidence, an effective skin anti-ageing protection, wound healing and scarring quality – all strongly depend on the sustainable mitochondrial functionality and well-balanced homoeostasis. The latter can be objectively measured and, if necessary, restored in a systemic manner by pre- and rehabilitation algorithms tailored to individualised patient profiles. The entire spectrum of corresponding innovations in the area includes natural and systemic skin rejuvenation, aesthetic and reconstructive medicine, sustainable skin protection and targeted treatments of skin disorders. Contextually, mitochondria-centric dermatology is instrumental for advanced 3PM-guided approach which makes a good use of predictive multi-level diagnostics and targeted protection of skin against both — the health-to-disease transition and progression of relevant disorders. Cost-effective targeted protection and new treatment avenues focused on sustainable mitochondrial health and physiologic homoeostasis are proposed in the article including in-depth analysis of patient cases and exemplified 3PM-guided care with detailed mechanisms and corresponding expert recommendations presented.

## Preamble

### Vitiligo as a prominent example of mitochondrial stress-related skin impairments

**Fig. 1:**
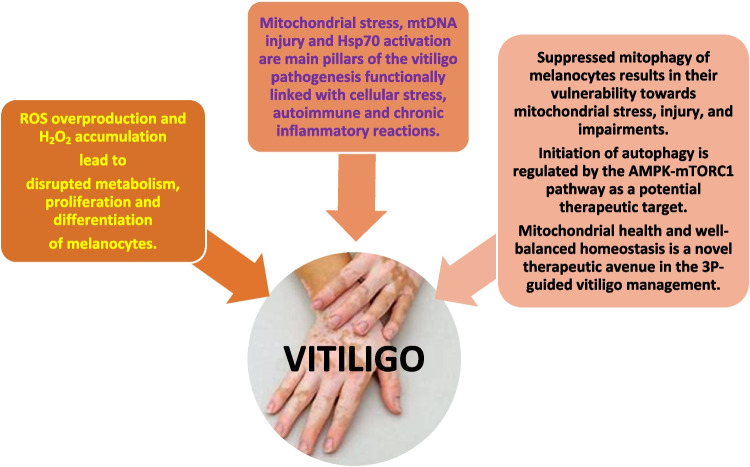
Impaired mitophagy promotes vulnerability of melanocytes towards mitochondrial stress, injury and impairments; new perspective for individualised predictive diagnostics and novel therapeutic pathways focused on mitochondrial health and homoeostasis in overall vitiligo management; AMPK, adenosine monophosphate-activated protein kinase; ATP, adenosine triphosphate; Hsp 70, heat shock protein 70; IL-17, interleukin 17; mtDNA, mitochondrial DNA; mTORC1, mammalian target of rapamycin complex 1; PI3K, phosphatidylinositol-3-kinase; ROS, reactive oxygen species

Vitiligo is a chronic autoimmune disease reflected in depigmented skin which is characterised by patches of discoloured skin on any part of the body [1]. Globally, it affects 0.5–2% of the population, regardless of age [2]. Vitiligo is defined by excessive death and/or impaired function of epidermal melanocytes. Although exact pathomechanisms of the disease-related melanocytes’ malfunction are largely unknown [3], immune system impairments and mitochondrial stress overload leading to the mitochondrial dysfunction are considered the main pillars of the excessive melanocytes’ death in the initiation of vitiligo [1, 3, 4], as summarised in Fig. 1. Per evidence, mitochondrial autophagosomes are absent in epidermal melanocytes within the active vitiligo lesions. In turn, suppressed melanocytes’ mitophagy promotes their vulnerability to mitochondrial stress, injury and impairments [4] — the discovery which opens new perspective for individualised predictive diagnostics, targeted disease prevention and novel therapeutic avenues focused on sustainable mitochondrial health and their well-balanced homoeostasis to advance cost-effective vitiligo management.

## Facts and hypotheses in the framework of 3P medicine

- Association of both extrinsic and intrinsic risk factors leading to accelerated skin ageing reflected in excessive ROS (reactive oxygen species) production and ir/reversible mitochondrial stress is abundantly reported.
- Maintained (primary care) and restored (secondary care) mitochondrial health may be critical to advance dermatological treatments, due to the key role of mitochondrial stress in the pathophysiology of skin disorders.
- The key role of physiologic mitochondrial homoeostasis and rejuvenation in maintaining healthy mitochondrial populations is considered to protect the skin against health-to-disease transition.
- Mitochondria-centric dermatology is instrumental for the 3PM-guided primary (cost-effective protection against health-to-disease transition) and secondary (protection against disease progression) care.

## The health of skin and mitochondria goes hand-in-hand

The skin is the largest organ of the human body with comprehensive structure and functions, one of which is protective against extrinsic stressors and invasions. To perform its complex function, skin possesses a unique cellular structure. Epidermis constitutes the upper layer of the skin, characterised by stratified structures covered with hair follicles. The outer layer of the epidermis is composed of cornified keratinocytes or corneocytes building up a cytoskeletal network of keratin filaments. Further, epidermal stem cells and progenitor cells reside in the basal layer, regenerating the epidermis continuously. Located below the epidermis, the dermis houses the greatest cell diversity within the skin compartments. Dermal fibroblast as the dominant cell type of the dermis are crucial for constructing and maintaining the extracellular matrix (ECM) consisting mainly of collagen, elastin, glycoproteins and glycosaminoglycans, which is fundamental to the skin’s elasticity and strength. The hypodermis resides beneath the dermis, mostly made up of adipocytes, pre-adipocytes, and adipose-derived stem cells anchoring skin to muscles and acting as a long-term repository of energy [5, 6].

Mitochondria are double-membrane organelles with a maternally inherited autonomous genome [6]. In the skin, mitochondria play a crucial role in cell signalling, wound healing, pigmentation, vasculature homoeostasis and hair growth [7]. Skin pathologies are associated with mitochondrial stress, injury and dysfunction [8]. Mitochondria play a vital role in microbial defence and protective function of skin against microbial invasions: an increased ATP production has been documented in response to hypoxia-induced metabolic stress caused by skin infections [7]. The skin is the organ characterised by high dynamics and cellular turnover that is reflected in particularly high energy consumption [9]. Oxidative stress and excessive ROS production are the main attributes of mitochondrial dysfunction highly relevant for skin disorders [10]. Finally, physiologic calcium uptake during mitochondrial respiration directly influences the quality of keratinocyte differentiation: adverse effects in the population of keratinocytes are observed by inhibited mitochondrial calcium uptake [6, 9].

Due to its function as the protective organ of the human body, the skin is permanently exposed to a variety of environmental stressors including UV light, active and passive cigarette smoking, low and high temperatures as well as air pollution, amongst others. Contextually, the skin experiences an extensive pressure of both extrinsic and intrinsic factors of ageing, resulting in gradual loss of skin functionality and its greater vulnerability towards patho-flora and disease triggers [11, 12]. Both skin and its mitochondria are injured by stressors and excessive ROS production followed by a gradual reduction in mitochondrial activity and formation of senescent cells [12, 13]. To this end, genes encoding complexes I, IV and V of the mitochondrial respiratory chain are frequently deleted in elderly people that is pronounced in the skin of individuals older than 70 years of age and high UV-exposure [14]. In turn, skin rejuvenation can be achieved by restoring mitochondrial function [15]. Aged skin is characterised by deeper wrinkles, rough texture and coarse appearance as well as by an uneven pigmentation [12, 16]. Chronic UV exposure damages mtDNA and induces oxidative stress in skin cells, giving rise to the photo-ageing and causing downregulation of collagen biosynthesis [6, 17–19]. Molecular mechanisms behind skin ageing include mitochondrial and chromosomal DNA damage, ROS overproduction, telomere shortening, chronic inflammation and altered molecular signalling pathways [20].

## Mitochondrial biogenesis and dynamic homoeostasis

Mitochondria are the most dynamic organelles within a cell undergoing physiologic homoeostasis which aims at maintaining healthy mitochondrial population. This includes biogenesis, fission, fusion and mitophagy which are synergistically regulated [21].

Mitochondrial biogenesis increases the population of healthy mitochondria in cells, also triggered by signalling cascades under stress conditions [22]. PPARγ co-activator 1 alpha (PGC-1α) plays a central role in mitochondrial biogenesis. Irradiation of human immortalised keratinocyte cells with UVB leads to a reduction in AMPK phosphorylation and significant downregulation of SIRT-1 and PGC-1α [23]. Per evidence, higher PGC-1α level boosts mitochondriogenesis and maintains physiologic mitochondrial functionality, thereby protecting the skin against ageing [24].

Mitochondrial fission process splits mitochondrial contents during cell division into two daughter mitochondria as well as enables damaged mitochondria to be eliminated by asymmetric fission [21]. Mitochondrial fusion facilitates the exchange of mitochondrial material as well as calcium and ROS buffering and generates larger mitochondrions. Together, these mechanisms are referred to as mitochondrial dynamics [25], which are implicated in cellular and mitochondrial repair pathways and maintain cellular and mitochondrial architecture (shape, size and distribution) under physiologically controlled processes [21, 23]. In contrast, impaired mitochondrial dynamics contribute to metabolic imbalance and ageing-related systemic diseases including skin pathologies [21]. To this end, an association between abnormal mitochondrial dynamics and dermatological symptoms of systemic diseases has been documented. This makes mitochondrial homoeostasis to an attractive target for the anti-ageing therapy which can be exemplified with the dynamin-related protein 1 expression relevant for the mitochondrial fission and mitophagy in cells [26, 27].

**Fig. 2:**
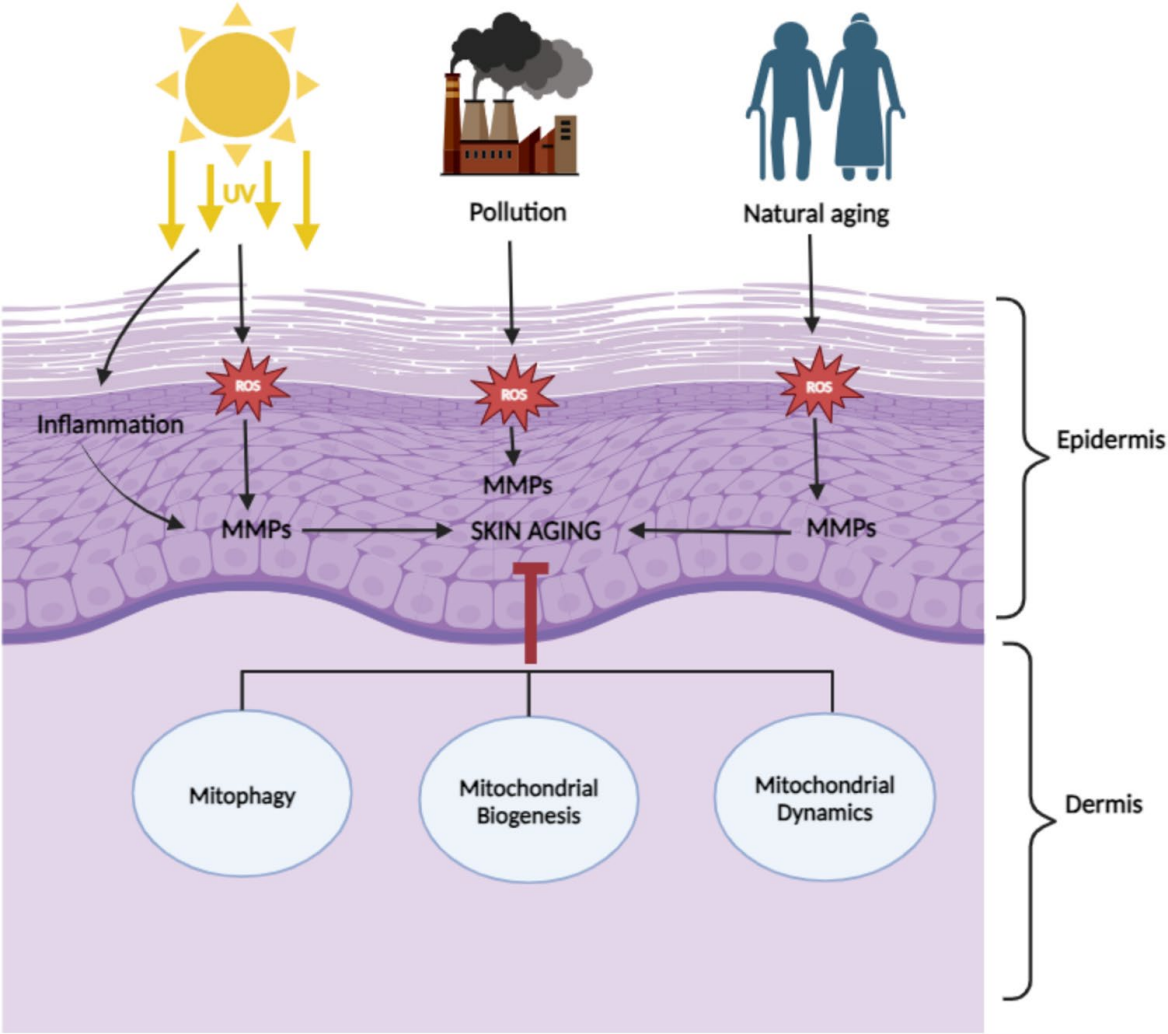
Physiologic (primary care) and restored mitochondrial homoeostasis is instrumental for reducing skin ageing risks and follow-up disorders caused by internal and external stressors. MMPs, matrix metalloproteinase; ROS, reactive oxygen species

To preserve mitochondrial quality, a selective type of autophagy, known as “mitophagy”, becomes activated to safeguard cells [21]. When cellular stress overburdens the mitochondrial quality control, excessively damaged mitochondria become eliminated through mitophagy [23] which frequently avoids activation of the basal autophagy, therewith protecting cells against apoptosis [28]. In this elegant manner, an increasing damage to skin cells is partially mitigated by blocking ROS overproduced in injured mitochondria. Mitophagy is known to be triggered by several complementary mechanisms capabale to compensate each other, at least partially, demonstrating, therefore, the critical role of mitophagy in physiologic homoeostasis of healthy mitochondria population [28]. Figure 2 demonstrates the key role of physiologic and restored mitochondrial dynamics, biogenesis and homoeostasis in primary and secondary care of skin physiology.

## Physiologic wound healing as a prominent example of the key regulatory role of mitophagy

The physiologic wound healing is a continuous and multifaceted process composed of sequential steps: haemostasis, inflammation, proliferation followed by the tissue remodelling and scar formation. The overall quality of all single steps performed in this sequence essentially depends on the intact energy metabolism, healthy mitochondrial populations and plenty of molecular pathways concerted in a holistic way [29–31]. Contextually, wound healing impairments result from systemic health adverse effects caused by metabolic disorders and vascular dysregulation, amongst others. Phenotyping of non-physiologic wound healing and stratification of affected patient cohorts and individuals in suboptimal health predisposed to these deficits are essential for both — primary (protection against health-to-disease transition) and secondary (protection against disease progression) care [32–36]. To this end, an adequacy of the mitophagy level is essential for each stage of the physiologic wound healing [37, 38]. In contrast, a dysregulated basal autophagy and mitophagy may result in chronic wounds and exaggerated ECM deposition scarring [39]. The mitophagy contributes to each phase of wound healing as detailed below.

1. Haemostasis stage: preservation of mitochondrial quality and platelet activation; mitochondrial membrane receptors such as BCL2-interacting protein 3-like (BNIP3L/NIX) play an integral role in mitophagy regulation [37, 40]
2. Inflammatory stage: a key regulatory role; for example, caspase 1 and 11 deactivate PTEN-induced kinase (PINK1)/Parkin and prevent mitophagy to stimulate the generation of mtROS, which trigger macrophage activation [41]; during the later inflammatory phase, interleukin-10 blocks the mammalian target of rapamycin (mTOR) signal to promote mitophagy, facilitating the conversion of macrophages from M1 to M2 state, which supports tissue regeneration [37, 42]
3. Proliferative stage: keratinocyte differentiation depends on NIX-mediated mitophagy [43]
4. Remodelling stage: boosting angiogenesis and tissue regeneration as well as stimulating wound healing signalling mechanisms via mitophagy [37]

Further, the critical role of the adequately regulated mitophagy in physiologic healing of burn injuries is well documented in the literature [37, 44]. According to the field-dedicated studies, after burn injuries, mitophagy is significantly elevated as confirmed by upregulated PINK1, Parkin, MFN1 and ubiquitin-specific protease [37, 45]. Mitophagy-targeting pharmaceutical interventions may prove to be a promising therapeutic strategy for burn injuries. To this end, deferiprone — the drug which acts as mitophagy trigger — demonstrates a great capacity reducing oxidative stress by 95% in burn injury models [46].

## Mitochondrial targeting is instrumental for maintaining skin health and triggering rejuvenation processes

Accumulated research data demonstrate positive effects of caloric restriction on the skin quality associated with stimulated mitochondrial quality and function in the dermis. As the result, epidermal thickens and hair follicle stem cell pools are increased that collectively reflect the skin rejuvenation processes [47]. In contrast, preclinical studies showed that mice lacking mitochondrial matrix superoxide dismutase-2 demonstrate an ageing phenotype with accelerated senescence of the skin cells [47].

Further, the majority of therapeutic agents prescribed for treating skin disorders primarily target mitochondrial function which dermatologists are not always aware of [9]. Generally, there are two major therapeutic approaches targeting mitochondria in the skin locally and systemically, namely (A) stimulating ATP production and (B) scavenging excessive ROS and therefore protecting mitochondria against injury [9]. Below listed natural substances are considered highly supportive for mitochondrial health and homoeostasis utilising both mechanisms A and B.

### Coenzyme Q10

Coenzyme Q10 (CoQ10), a lipophilic isoprenylated quinone, is a natural ROS scavenger, serving as an electron shuttle between Complexes I/II and III of the electron transport chain [9, 48]. The existence of CoQ10 in eukaryotic cells’ membranes implies its potential as an antioxidant and free radical scavenger [9, 38]. CoQ10 is particularly accumulated in tissues with high energy demands like the heart, skeletal muscles, and neurons, which highlights its critical bio-energetic function [49]. CoQ10 levels decrease gradually with age [50]. Besides endogenous synthesis, CoQ10 is also obtained from exogenous sources like meat, fish, chicken legs, nuts and trout [49]. CoQ10 deficiencies may result from both genetic and epigenetic deficits leading to excessive oxidative stress and accelerated ageing [38, 49]. Physiologically, CoQ10 is an essential part of both skin cells and skin surface lipids (SSL), a component of the stratum corneum, where it functions together with other molecules as the skin’s primary protective shield against oxidant damage [51, 52]. There is a strong association between anti-ageing skin protection and CoQ10’s antioxidant and bio-energetic properties [6]. Contextually, the significantly reduced activity of the complexes I/III and II/III as well as excessive generation of superoxide anions are consistent with diminished CoQ10 content in aged dermal fibroblasts [53]. CoQ10 is widely employed in skin care cosmetics to alleviate ageing signs and to protect the skin from health adverse effects of free radicals. A number of studies were able to show that topically applied CoQ10 significantly reduces ageing symptoms of the photo-aged skin by restoring mitochondrial functionality [54] and increasing the collagen density [55]. Also, age-related ROS production in keratinocytes along with loss of mitochondrial membrane potential leading to anaerobic glycolysis is associated with impaired CoQ10 metabolism [56]. Furthermore, applying CoQ10 on the skin for 3 months resulted in reduction of wrinkle extent. As a result of topical CoQ10 administration, the epidermal content is increased at SSL and in deeper layers of the epidermis. In addition, the skin’s antioxidant capacity is also elevated [57]. Aged skin cells were found to benefit from CoQ10 by accelerated restoration of cellular ATP levels achieved by enhanced mitochondrial functionality and minimised oxidative stress [58].

CoQ10 is also used in aesthetic medicinal products making a good used of its skin health–promoting properties. Further, vitamin E combined with CoQ10 and taken orally, has been shown to enhance skin CoQ10 levels systemically and to reduce wrinkle depth [51]. In one study involving 12 weeks of daily intake of a water-soluble form of CoQ10 with superior bioavailability (Q10Vital®), some visible signs of skin ageing were visibly reduced in a systemic manner. A significant improvement in the smoothness of the skin was also seen, as well as a reduction in wrinkles and micro-relief lines [38].

### Resveratrol

Resveratrol is one of the well-known polyphenols which has been studied extensively by the scientific community due to a wide range of its health-supportive biological functions, including antioxidant, anti-inflammatory, anti-obesity and anti-cancer protective properties [49, 59]. Resveratrol shields cells from ultraviolet-induced cell apoptosis and oxidative stress. These effects of resveratrol are partially explained by its ability to neutralise free radicals and to support antioxidant molecular pathways [60, 61]. Thus, protective effects of the mitochondria-specific antioxidant, resveratrol and the cellular antioxidant, curcumin, against UVA-induced mtDNA damage in human dermal fibroblasts underwent a comparative analysis which revealed strongly pronounced photo-protective effects of resveratrol [62]. Table 1 summarises research data towards stimulating effects of resveratrol regarding the wound healing quality, quality of scarring and protection against photo-ageing.

**Table 1:** Summary of resveratrol’s effects on wound healing, photo‐ageing and quality of scarring; *α‐SMA* α‐smooth muscle actin, *EGFR* epidermal growth factor receptor, *HSFBs* human hypertrophic scar‐derived fibroblasts, *HSP27* heat shock protein 27, *MMP-1* matrix metalloproteinase 1, *MMP-9* matrix metalloproteinase 1, *NHDFs* normal human dermal fibroblast cells, *Nrf2* nuclear factor erythroid 2-related factor, *ROS* reactive oxygen species, *Sirtuin 1* SIRT1, *VEGF* vascular endothelial growth factor

Positive effects measured	Mechanism	Reference
Anti‐inflammatory; wound healing supportive	• Interacting with EGFR‐controlled cytoplasmic and nuclear pathways	[63–67]
	• Stabilising cell proliferation, improving migration quality and ultrastructural preservation	
	• Inducing expression of VEGF in keratinocytes and regulating angiogenesis	
	• Activation of SIRT1	
	• Improving vascularization of the wound bed attributed to stimulation of AMPK pathway	
Photo-protective; anti-ageing protective	• Downregulating ROS overproduction in NHDFs following UVB exposure • Reducing MMP‐1 expression and transcription of inflammatory cytokines responsible for inflammageing • Increasing expression of type I collagen	[68–72]
	• Upregulating HSP27 expression	
	• Suppressing of UVB‐induced epidermal thickening, decreasing expression of MMP‐1 and MMP‐9 following oral and topical administration of resveratrol	
	• Activating Nrf2 pathway and reducing ROS production, supporting collagen type II alpha 1 gene expression	
	• Restoring natural water‐lipid layer and intercellular cement in skin deeper layers	
Scarring quality promoting	• Suppressing cell proliferation in HSFBs, resulting in cell cycle arrest and apoptosis induction in a dose‐ and time‐dependent way • Increasing SIRT1‐expression and reducing α‐SMA expression	[73, 74]

### Niacinamide

Niacinamide, a member of the vitamin B-family, is a water-soluble molecule that contributes to multi-faceted physiological mechanisms of mammalian cells essentially including energy metabolism [75]. This vitamin is composed of two molecular components, i.e. niacin or nicotinic acid and niacinamide or nicotinamide [49]. Niacinamide plays a key role in the synthesis of nicotinamide adenine dinucleotide (NAD +), which is then required for the synthesis of NADH and NADPH coenzymes. In mammalian cells, they are crucial for the redox balance and energy metabolism [76]. There is convincing evidence that niacinamide may promote cellular lifespan by engaging in DNA repair and alleviation of oxidative stress and inflammatory responses [75]. The antioxidant properties of niacinamide made it to a key excipient in dermo-cosmetic products for regenerating aged and sun-damaged skin [76]. There are several positive effects of a systemic niacinamide supplementation, including restoration of the cellular NAD + pool and mitochondrial energy levels, reduction of oxidative stress and inflammation, improvement of the extracellular matrix and skin barrier and suppression of an uneven pigmentation in the skin [58]. It is a widely used ingredient for rejuvenating aged and sun-damaged skin. Topical application of nicotinamide, alone or formulated with other effective agents, slows down skin ageing progression and has anti‐inflammatory effects against rosacea, acne, autoimmune skin pathologies and hyperpigmentation [6, 77].

### Quercetin

Quercetin is a polyphenolic flavonoid which is abundantly available in fruits (grapes, peaches) and vegetables (onions, garlic etc.) [78]. Accumulated knowledge demonstrates positive health effects of quercetin on anti-inflammatory cytokines and mitochondrial homoeostasis by targeting SIRT1 activity via the SIRT1/AMPK/NF-κB, SIRT1/Keap1/Nrf2/HO-1, and SIRT1/PI3K/Akt pathways [78]. Quercetin is one of the best acknowledged natural activators of mitophagy [38]. Quercetin effectively suppresses expression rates of atopic dermatitis-induced IL-1β, IL-6, IL-8, while supporting activities of superoxide dismutase-1, superoxide dismutase-2, catalase, glutathione peroxidase, and interleukin −10 [79]. Further, quercetin promotes wound healing by suppressing pro-inflammatory cytokines and upregulating transcription factors that regulate epithelial-mesenchymal transition [79, 80]. In aged human dermal fibroblasts, quercetin reduces intracellular and extracellular ROS levels, and supports mitochondrial functionality and rejuvenation [81].

## You cannot manage, if you cannot measure — case reports

This subchapter introduces selected patient cases to illustrate importance of the patient stratification, phenotyping and measurement of mitophagy e.g. in the tear fluid.

### Case report 1

A female patient with evident Flammer syndrome phenotype (FSP) demonstrating characteristic signs and symptoms which are indicative for predictive approach and targeted preventive measures and treatments tailored to individualised patient profiles.

General information: female, 24 years old, Caucasian, academic making a successful international scientific career, professionally active about 10 h daily; stress situations are frequent.

FSP survey has identified following specific symptoms and signs:

**Table:** 

Cold hands and/or feet	Yes	frequently even during summer time and particularly in stress situations
Feel cold	Yes	frequently
Low blood pressure	Yes	
Dizziness	Yes	e.g. by standing up and changing altitude
Prolong sleep onset	Yes	altered circadian rhythms
Do not feel thirsty	Yes	even in hot weather
Migraine with aura	Yes	strongly pronounced
Altered reaction towards drugs	Yes	strongly pronounced
Altered pain sensitivity	Yes	extremely pain sensitive
Strong smell perception	Yes	extraordinary pronounced
Slim at 20–30 years of age	Yes	BMI = 19 kg/m^2^
Tendency towards perfectionism	Yes	strongly pronounced
Tinnitus	Yes	with acutely reduced hearing in stress situations
Reversible skin blotches	Yes	strongly pronounced in stress situations
Slowed/impaired wound healing	Yes	strongly pronounced
Increased endothelin level	Yes	> 3.0 pg/ml in blood serum

At the age of 24 years, she gave birth to a child by Caesarean section. Pregnancy complications: oligohydramnios, pronounced nausea and vomiting during entire pregnancy which is considered characteristic for FSP carriers, potentially due to a reduced liquid intake and an increased sensitivity to metabolic and cardiovascular stress [82]. Caesarean section resulted in an enormous blood loss; the post-surgical recovery has taken several months being complicated with an impaired healing of the wound still bleeding a couple of months after the surgery performed.

The functional link between FSP, energy deficits, compromised mitochondrial health and potentially impaired wound healing is evidence-based and described in the peer-reviewed scientific literature [32, 34, 35, 83, 84].

Contextually, mitochondria-based holistic approach is strongly recommended for primary (protection against health-to-disease transition) and secondary (protection against disease progression) care of individuals with the FSP [38, 84–86]. Further, tear fluid is considered an optimal source of information for health risk assessment applied to the FSP carriers [38, 87].

Tear fluid analysis performed for the patient demonstrated a significantly decreased mitophagy level compared to the reference values in corresponding group of age (the know-how of **3PMedicon GmbH** performing internationally validated tests [88]; the methodology is described elsewhere [85]).

The patient observed chronic fatigue symptoms correlating well with the detected high level of mitochondrial stress.

Mitochondrial health-relevant nutraceuticals were recommended, which per scientific evidence are of great clinical utility for FSP carriers [38] to stabilise health condition of the patient and to improve wound healing, namely, CoQ_10_ (increases ECT efficacy, mitigates oxidative stress and fatigue, protects mitochondrial health), vitamins B1 and B2 (increases ETC efficacy), quercetin (promotes mitophagy), Mg (stimulates NO production and vasodilatation) and vitamin D3 (promotes wound healing).

### Case report 2

General information: female, 52 years old, BMI = 24 kg/m^2^, single, smoker, stressful daily job performance, middle socio-economic status, psychological distress which she daily deals with (post-stroke complications of her mother).

Health-related complains: chronic fatigue, sometimes anxiety and even depression, chronic systemic inflammation, skin allergy, symptoms of accelerated cutaneous ageing.

An association between the above presented medical conditions and compromised mitochondrial health is abundantly described in the peer-reviewed scientific literature [31, 89–98].

Tear fluid analysis performed for the patient demonstrated a significantly decreased mitophagy level compared to the reference values in corresponding group of age (the know-how of **3PMedicon GmbH** performing internationally validated tests [88]; the methodology is described elsewhere [85]).

Contextually, mitochondrial component should be essentially considered to mitigate systemic effects which are functionally linked with dermatological symptoms.

Mitochondrial health-relevant nutraceuticals, which per scientific evidence are recommended for patients with above listed symptoms [38] to stabilise her health condition, are the following ones: CoQ_10_ (increases ECT efficacy, mitigates oxidative stress and fatigue, protects mitochondrial health), quercetin (promotes mitophagy and mitochondrial and cellular rejuvenation), resveratrol (systemic anti-inflammatory effects).

## Conclusions and expert recommendations in the framework of 3PM

Association of both intrinsic and extrinsic risk factors leading to accelerated skin ageing reflected in excessive ROS production and ir/reversible mitochondrial stress is abundantly demonstrated in scientific literature. Contextually, maintained (primary care) or restored (secondary care) mitochondrial health and homoeostasis are considered powerful instruments to advance dermatological treatments including aesthetic medicine, due to the critical role of mitochondrial stress in the pathophysiology of skin ageing and disorders. Mitochondria-centric dermatology is instrumental for the advanced 3PM-guided approach based on predictive diagnostics and cost-effective targeted protection of skin against both health-to-disease transition and disease progression. New therapeutic avenues focused on sustainable mitochondrial health and physiologic homoeostasis, are proposed in the article. Table 2 summarises health conditions linked to compromised mitochondrial health and dermatologic impairments with underlying mechanisms and proposed evidence-based 3PM-guided care.

**Table 2:** Prominent conditions relevant for both — compromised mitochondrial health and dermatologic impairments; mitochondrial health quality check-up is essential to predict disease development and progression, to apply targeted prevention and treatments tailored to the person as well as to monitor treatment efficacy — altogether a multi-modal 3PM-guided approach; corresponding references are presented

Conditions linked to compromised mitochondrial health and dermatologic impairments	Functional links; Underlying mechanisms; 3PM-guided care	References
**Extrinsic environmental risks and risks linked to professional occupation**		
Toxic environment: - Toxic substances - Heavy metals - Abnormal air pollution - Abnormal pH, osmolarity and temperature of water etc.	Mitochondrial stress; damaged mtDNA and chrDNA; challenged repair machinery; highly increased energy demand; potential “vicious circle” leading to mitochondrial burnout; progression from health to autoimmune pre-disease with skin manifestation - Cutaneous vulnerability - Atopic dermatitis - Cutaneous irritancy of water - Mitochondrial sirtuins in skin - Adequate cutaneous response to environmental stressors - Mitophagy	[19, 31, 89, 92, 99, 103]
Exposure to ionising/UV irradiation: geo-specific natural irradiation; artificial contamination; long and frequent flights; solar insults	Mitochondrial stress; challenged repair machinery; highly increased energy demand; potential “vicious circle” leading to mitochondrial stress and burnout with skin manifestations: - Cutaneous vulnerability - Photo-carcinogenesis - Mitochondrial sirtuins in skin - Adequate cutaneous response to environmental stressors Protective effects by restored physiologic mitochondrial homoeostasis and efficient cellular bioenergetics	[19, 31, 89, 92, 104]
Non-physiologic timeframe of job performance and shift-work	Shifted circadian rhythms; circadian misalignment; compromised sleep quality; mitochondrial stress and burnout; progression from health to autoimmune pre-disease with skin manifestation - Multi-faceted effects of hormonal and stress mediators on skin innate and adaptive immunity - Skin autoimmune diseases - Circadian disruption as a risk of melanoma Protective effects of melatonin on mitochondria and skin	[31, 89, 103, 105, 106]
**Socio-economic and life-style associated risks**		
Malnutrition: imbalanced and deficient dietary patterns	Preventable mitochondrial impairments related to malnutrition; preventable skin impairments; progression from health to autoimmune pre-diseases - Compromised skin structure and functions - Affected skin microbiome	[38, 103, 107, 108]
**Genetic risks**		
Family predisposition to chronic severe pathologies; genetic diseases	Hereditary genetics reflected in non-modifiable risks of metabolic shifts, cardiovascular, malignant and neuro/degenerative pathologies, amongst others — all relevant to compromised mitochondrial health Skin manifestations: cutaneous vulnerability, ageing and diseases	[99, 109, 110]
**Exemplified relevant medical conditions**		
Vascular status associated conditions	Increased endothelin-1 level as persistent stressor; increased vascular stiffness, primary and secondary vasospasm, low and high blood pressure, ischemia–reperfusion, Flammer syndrome phenotype, etc. — all linked to mitochondrial damage, decreased repair capacity and cutaneous depressions	[31, 91, 111, 113]
Psychological distress	Decompensated mitochondrial stress and potential mitochondrial burnout; adverse systemic effects to health with skin manifestations - Cutaneous vulnerability - Skin ageing	[31, 89, 93, 94]
Burnout syndrome		
Chronic fatigue		
Abnormal sleep patterns		
Inadequate stress response/behavioural patterns		
Psycho-trauma and post-traumatic stress disorder		
Frequent acute infections	Extensive mitochondrial injury; adverse systemic effects to health; - Cutaneous vulnerability - Skin microbiome — implication for wound care	[90–93]
Chronic inflammation and associated conditions		
Prolonged/impaired wound healing		
Microbiome shifts, pathogenic bio-flora and maintenance of skin barrier function	Mitochondrial stress is highly indicative for systemic effects caused by bio-toxins; relevant sources of information: body fluids and skin	[90, 101, 114]
Allergies	From allergy to autoimmunity; multi-faceted involvement of mitochondria; systemic effects - Skin irritation - Skin microbiome - Atopic dermatitis	[95–98]
Accelerated ageing	Higher biologic age against chronologic age — decreased mitochondrial functionality; systemic effects with skin manifestations: - Skin is exposed to daily environmental insults - Cutaneous vulnerability - Cutaneous depressions Restored physiologic mitochondrial homoeostasis and efficient cellular bioenergetics with improved individual outcomes	[93, 104, 115, 116]
**Mitophagy-based check-up recommended for mitochondria-centric dermatological approaches**		
Regular health status check-up in primary care: cost-effective prevention of health-to-disease transition	Suboptimal health status; reversible damage to health; multi-factorial risks with skin manifestations: cutaneous vulnerability and cutaneous redox senescence Assessed mitophagy dynamics and restored physiologic mitochondrial homoeostasis and efficient cellular bioenergetics with improved individual outcomes	[38, 117]
Regular health status check-up in secondary care: patients after treated disorders (acute viral and bacterial infections, malignancies etc.); patients diagnosed with metabolic syndrome, CVDs and degenerative diseases, etc.	Disease- and treatment-associated ageing; regeneration stimulating mechanisms; cutaneous vulnerability; cutaneous depressions; cutaneous redox senescence Assessed mitophagy dynamics and restored physiologic mitochondrial homoeostasis and efficient cellular bioenergetics with improved individual outcomes	[38, 93, 118]
Anti-ageing programmes	Holistic approach; systemic effects; regeneration stimulating mechanisms: - Cutaneous rejuvenation - Improved individual outcomes	[38, 93, 118]
Reconstructive and aesthetic surgery	Complex pre- and rehabilitation programmes: - Predictable scar quality - Cutaneous rejuvenation - Optimal individual outcomes	[38, 82, 118]

## Data Availability

No datasets were generated or analysed during the current study.

## Abbreviations

chrDNA: Chromosomal DNA
CoQ10: CoenzymeQ10
ECM: Extracellular matrix
FSP: Flammer syndrome phenotype
MMPs: Matrix metalloproteinases
mtDNA: Mitochondrial DNA
OXPHOS: Oxidative phosphorylation
PGC-1α: PPARγ co-activator 1 alpha
PINK1: PTEN-induced kinase
ROS: Reactive oxygen species
SIRT1: Sirtuin 1
SSL: Skin surface lipids
